# Australasian sky islands act as a diversity pump facilitating peripheral speciation and complex reversal from narrow endemic to widespread ecological supertramp

**DOI:** 10.1002/ece3.517

**Published:** 2013-03-07

**Authors:** Emmanuel F A Toussaint, Katayo Sagata, Suriani Surbakti, Lars Hendrich, Michael Balke

**Affiliations:** 1 Zoological State CollectionMünchhausenstraße 21, Munich, 81247, Germany; 2 Papua New Guinea Institute for Biological research (PNG-IBR)Goroka, Papua New Guinea; 3 Jurusan Biology, FMIPA-Universitas CendrawasihKampus Baru, Jayapura, Papua, Indonesia; 4 GeoBioCenter, Ludwig-Maximilians-UniversityMunich, Germany

**Keywords:** Australian region, diversity pump, highlands, New Guinea, New Zealand, peripheral speciation

## Abstract

The Australasian archipelago is biologically extremely diverse as a result of a highly puzzling geological and biological evolution. Unveiling the underlying mechanisms has never been more attainable as molecular phylogenetic and geological methods improve, and has become a research priority considering increasing human-mediated loss of biodiversity. However, studies of finer scaled evolutionary patterns remain rare particularly for megadiverse Melanesian biota. While oceanic islands have received some attention in the region, likewise insular mountain blocks that serve as species pumps remain understudied, even though Australasia, for example, features some of the most spectacular tropical alpine habitats in the World. Here, we sequenced almost 2 kb of mitochondrial DNA from the widespread diving beetle *Rhantus suturalis* from across Australasia and the Indomalayan Archipelago, including remote New Guinean highlands. Based on expert taxonomy with a multigene phylogenetic backbone study, and combining molecular phylogenetics, phylogeography, divergence time estimation, and historical demography, we recover comparably low geographic signal, but complex phylogenetic relationships and population structure within *R. suturalis*. Four narrowly endemic New Guinea highland species are subordinated and two populations (New Guinea, New Zealand) seem to constitute cases of ongoing speciation. We reveal repeated colonization of remote mountain chains where haplotypes out of a core clade of very widespread haplotypes syntopically might occur with well-isolated ones. These results are corroborated by a Pleistocene origin approximately 2.4 Ma ago, followed by a sudden demographic expansion 600,000 years ago that may have been initiated through climatic adaptations. This study is a snapshot of the early stages of lineage diversification by peripatric speciation in Australasia, and supports New Guinea sky islands as cradles of evolution, in line with geological evidence suggesting very recent origin of high altitudes in the region.

## Introduction

Deciphering the mechanisms of species formation is one of the most fascinating and challenging areas of evolutionary biology (Darwin and Wallace 1858; Darwin 1859; Mayr and Diamond 2001; Coyne and Orr 2004; Fitzpatrick et al. 2009; Santini et al. 2012). Many studies on biogeographic and ecological factors promoting speciation have helped to establish the separation by physical barriers (“vicariance”) followed by genomic isolation as the null hypothesis that begets new species (Lynch 1989; Barraclough and Vogler 2000; Mayr and Diamond 2001; Johannesson 2010; Santini et al. 2012). However, although restriction of gene flow and allopatric speciation appear to be the most common mechanisms, different processes have also been documented through the years (White 1968; de Aguiar et al. 2009; Johannesson 2010). For instance, sympatric speciation, suggested already by Darwin (1859) before being tested and supported for many metazoan taxa (e.g., Crow et al. 2010), implies that speciation events can occur within the same population through genetic polymorphism. Furthermore, special cases of allopatric speciation are found in parapatric and peripatric models, which invoke processes at the distributional periphery of an ancestral species, where individuals might enter a new habitat most likely facilitated by divergent ecological characteristics. In this case of peripheral speciation, also known as “budding speciation,” either the geographic isolation, ecological factors, or a combination of the two leads to a cessation of gene flow, thus enabling the speciation process (Mayr 1982; Fitzpatrick and Turelli 2006).

Here, we focus on the Australasian archipelago and surrounding areas, a region that shelters a rich yet highly threatened biodiversity; seven biodiversity hotspots are situated in the Indomalayan-Australasian region (Mittermeier et al. 2004; conservation.org). This region, despite its highly complex geological history (e.g., Hall 2011; Metcalfe 2011), represents an ideal laboratory to study lineage diversification and speciation (Wallace 1860; Mayr and Diamond 2001; Condamine et al. 2013). Thousands of islands, many of them scattered across the Equator, varying in size from tiny patches to continental sized landmasses, ranging from young to geologically old and low-lying to high altitudes including snow-capped summits, harbor hyperdiverse biota and exceptional radiations. Particularly across the megadiverse Wallacea and Melanesia, most studies to date have investigated larger scale evolutionary patterns, whereas factors promoting speciation remain scarcely addressed, and the evolutionary processes involved are little known despite increased recent efforts (e.g., Von Rintelen et al. 2004; de Bruyn and Mather 2007; Joseph and Omland 2009; Craft et al. 2010; Deiner et al. 2011; Klaus et al. 2013). A unique feature in the Indomalayan-Australasian archipelago is its long chain of islands often with high mountains, usually surrounded by tropical lowland rain- or dry-forest. “Sky island” ecosystems are isolated patches surrounded by dramatically different lowland ecosystems (Heald 1967), in this case, isolated further from each other by ocean. Recently, such highland ecosystems have been shown to act as evolutionary cradles shaping a flourishing biota in diverse regions of the World (Hall 2005; Smith and Farrrell 2005; Robin et al. 2010; Schultheis et al. 2012). In the Australasian region, sky islands are geologically young (<5 Mya; Cloos et al. 2005), yet highly diverse (e.g., Mittermeier et al. 2004). Numerous studies on Australian and New Zealand mountain ranges have investigated speciation patterns and indicate important vicariant effects from mountain uplift per se, and climate change as further promoter of species diversity (e.g., Trewick et al. 2000; McCulloch et al. 2010; Hawlitschek et al. 2012). The mountains of Indonesia and northern Australasia show especially striking altitudinal gradients, usually being surrounded by tropical lowland rain- or dry-forest. Within this biodiversity-rich assemblage of mountainous ecosystems, one of the largest and most remote highland regions is the central New Guinean cordillera, with vast expanses of tropical montane and subalpine habitat, and extensive areas above 3500-m altitude, including numerous summits above 4500 m. Their role as a diversity pump for the archipelago remains poorly studied and little appreciated, despite their vast geographic extent and extreme structuring (but see Mayr and Diamond 1976).

**Figure 1:**
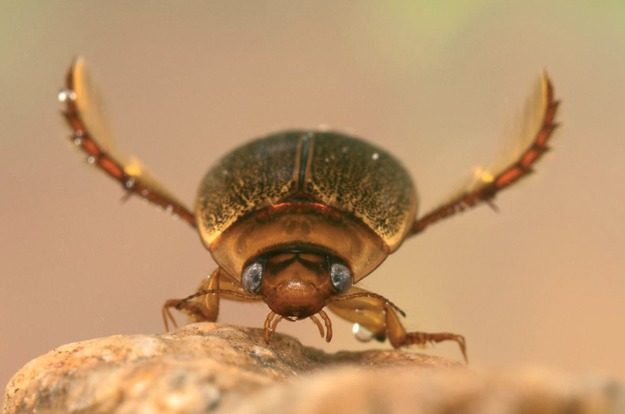
Habitus of *Rhantus suturalis* (Photo credit: Jan Hamrský)

**Figure 2:**
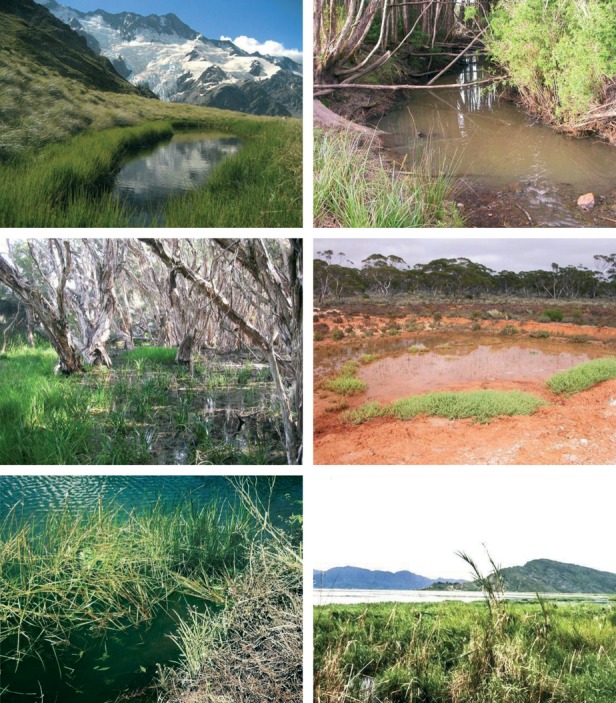
Habitats and habitus of the *Rhantus suturalis* southern clade in Southeast Asia and Australasia. Top left: montane peatland pond in Sealy Tarns (∼1300 m) (New Zealand); top right: Welcome River in the North-West of Tasmania; center left: flooded paperbark swamp in Beeliar wetlands (Western Australia); centre right: temporary pool in the Mallee near Balladonia (Western Australia); bottom left: edge of a lake in Ranu Pani (East Java); bottom right: Lake Paniai (1700–3000 m) sheltering *R. ekari* in West Papua.

**Figure 3:**
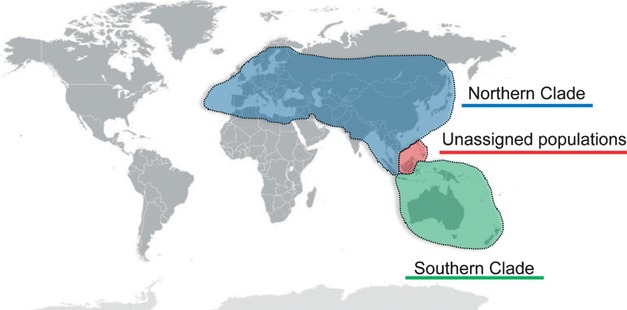
Distribution of *Rhantus suturalis*. Sequence data were not available for the area in red.

Here, we study pond-dwelling *Rhantus* diving beetles, often abundant in tropical montane and subalpine pond habitats across the Australasian region. There are at least 30 endemic species in the region including Oceania, mostly narrow endemics restricted to a single high valley, or few mountain tops. There is one striking exception, however: *Rhantus suturalis* MacLeay, 1825 (Coleoptera, Dytiscidae, Colymbetini) (Fig. 1), is a widespread ecological supertramp, ranging from the Azores islands to New Zealand, and inhabits diverse lentic habitats in mountainous or subalpine environments, for example, high altitude lakes (Fig. 2) and highland peat swamps (with pH around 4–5). It occurs in temperate lowland swamps, many anthropogenic habitats (freshly dug fish ponds, reservoirs, roadside ditches, cattle troughs), saline desert wetlands in North African deserts, and many more. Along with its closer relatives, it has never been found in tropical lowlands (Balke 1993, 2001; Balke et al. 2009). *Rhantus suturalis* is often an early colonizer of newly available habitats, hence referred to as a “supertramp species” (Balke et al. 2009). It is an ecological generalist, with high physiological tolerance, for example, in terms of salinity, temperature, and acidity. A comprehensive molecular phylogeny of *R. suturalis* from across its wide range revealed two major clades – a northern one, from Portugal to Sumatra, and a southern one from adjacent Java eastward to New Caledonia (Fig. 3) (Balke et al. 2009). Both *R. suturalis* clades contain one or more narrow-endemic species previously described based on marked morphological divergence. This species paraphyly was supported by extensive mitochondrial and nuclear DNA sampling (>7000 bp). A recent origin of *R. suturalis*, *c*. 6.0–2.7 Ma ago, was suggested, possibly in New Guinea, followed by an ancestral colonization of the Malay Archipelago and a large part of the Australian region. The rise of a widespread generalist out of a clade of narrow endemics not only refutes the assumption of “specialisation as an evolutionary dead-end” (Mayr 1963; coined by Cope 1896 “the law of the unspecialized”), but offers an opportunity to study the early phases of lineage diversification across a wide species range, which is nevertheless constrained by climate and other ecological factors.

In this study, we use extensive sampling across the Indomalayan-Australasian region to (1) reconstruct phylogenetic relationships within the southern clade of the widespread *R. suturalis*, (2) investigate phylogeographic patterns using haplotype network inferences, (3) infer the historical demography and timing of divergence of this group in a paleoclimatic framework, in order to test the hypothesis of ongoing peripatric speciation in Australasia, particularly New Guinea, sky islands, and (4) examine whether those mountain chains act as a species pump in a “cradle of evolution” model, or as an ancient biotic pool, in a “museum” model.

## Materials and Methods

### Taxon sampling and molecular biology

**Figure 4:**
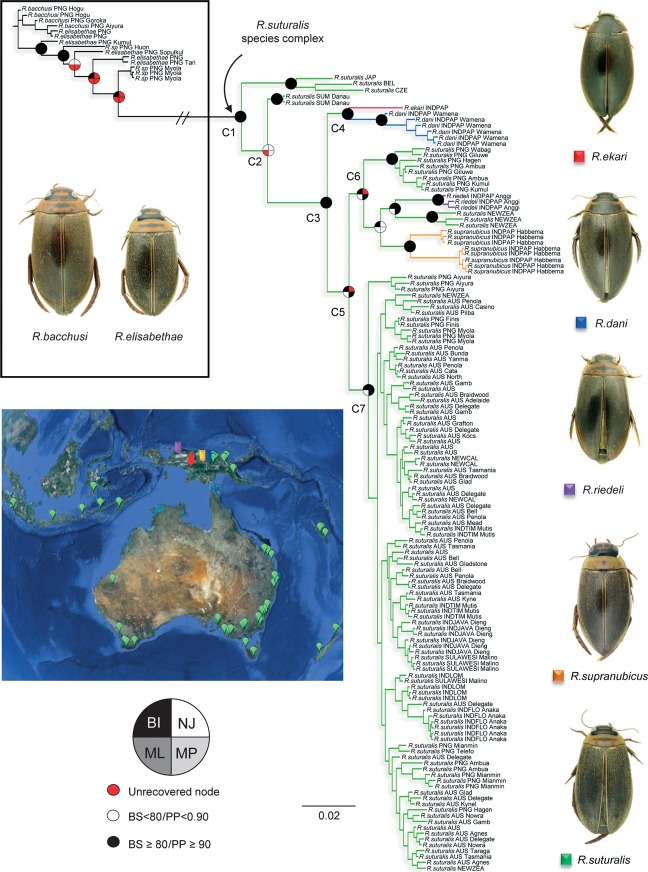
Phylogenetic relationships of the *Rhantus suturalis* species complex *Combined* dataset with the best-fitting strategy of partitioning under Bayesian Inference. Supports for each node are indicated according to the caption inserted in the figure (BI, Bayesian inference; NJ, neighbor-joining; MP, maximum parsimony; ML, maximum likelihood). A map highlighting collection localities is shown, in which the colors of the spots refer to the respective colored squares underneath the habitus of the different species (e.g., *R. riedeli* in purple). The major clades are labeled C1 to C7. Names of the species for which a habitus is displayed are specified under the pictures.

**Figure 5:**
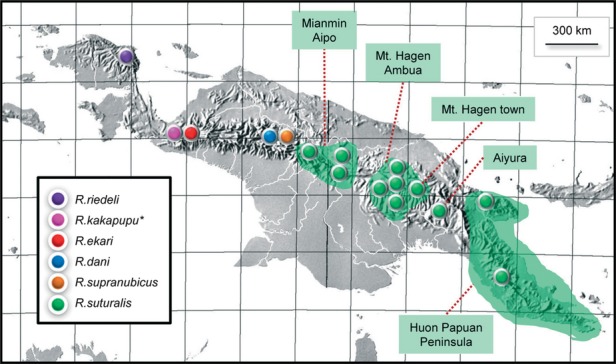
Distribution of New Guinean endemic species of the *Rhantus suturalis* complex. The different colors refer to the distribution of each species except *R. suturalis* for which they refer to the sampling localities (the distribution of *R. suturalis* in New Guinea is given by the green areas). The correspondences of the colors are shown in the legend at the bottom left corner of the figure. The asterisk indicates that *R. kakapupu* was not included in this study.

We sequenced 133 individuals of *R. suturalis* from 12 regions across the range of the southern clade (Table 1, Fig. 4) including the Sunda Islands, almost the entire New Guinean highland chain, Australia, New Zealand, and New Caledonia. *Rhantus suturalis* is known from old Philippine (Baguio) as well as Malaysian Borneo (Mt. Kinabalu) specimens (Balke 1993), but we did not manage to obtain fresh samples. We sampled most of the New Guinean endemic species (Fig. 5) subordinated within *R. suturalis*, that is, (1) *Rhantus dani*, Balke 2001 (usually shaded wetlands in an isolated montane depression, the Baliem Valley ∼1700 m), (2) *Rhantus ekari*, Balke and Hendrich 1992 (swampy edge of a large montane lake, Lake Paniai ∼1900 m) (Balke and Hendrich 1992), (3) *Rhantus riedeli*, Balke 2001 (same habitat but at Lake Anggi ∼1900 m), and (4) *Rhantus supranubicus*, Balke 2001 (alpine peat swamp pools and edge of Lake Habbema as well as Mount Elit swampland, ∼3300 m) (Balke 2001). *Rhantus kakapupu,* Balke 2001, described from old specimens collected across Lake Paniai where *R. ekari* occurs, was not found recently.

**Table 1:** Collecting localities, with code and number of specimens

Species	Country	Region	Code	Locality	Specimens
*Rhantus bacchusi*	Papua New Guinea	Eastern Highlands	PNGEHP	Aiyura	1
*R. bacchusi*	Papua New Guinea	Eastern Highlands	PNGEHP	Goroka	1
*R. bacchusi*	Papua New Guinea	Eastern Highlands	PNGEHP	Hogu	2
*R. dani*	Indonesia	Papua	INDPAP	Wamena	6
*R. ekari*	Indonesia	Papua	INDPAP	Enarotali	1
*R. elisabethae*	Papua New Guinea	Enga	PNGENG	Mt. Hagen Kumul Lodge	3
*R. elisabethae*	Papua New Guinea	Southern Highlands	PNGSHP	Mt. Giluwe Sopulkul	1
*R. elisabethae*	Papua New Guinea	Southern Highlands	PNGSHP	Tari	2
*R. riedeli*	Indonesia	Papua	INDPAP	Anggi	3
*Rhantus* sp.	Papua New Guinea	Central	PNGCEN	Myola	3
*Rhantus* sp.	Papua New Guinea	Morobe	PNGMOR	Huon	1
*R. supranubicus*	Indonesia	Papua	INDPAP	Lake Habbema	8
*R. suturalis*	Australia	New South Wales	AUSNSW	Bellingen	3
*R. suturalis*	Australia	New South Wales	AUSNSW	Braidwood	3
*R. suturalis*	Australia	New South Wales	AUSNSW	Casino	1
*R. suturalis*	Australia	New South Wales	AUSNSW	Delegate	9
*R. suturalis*	Australia	New South Wales	AUSNSW	Grafton	1
*R. suturalis*	Australia	New South Wales	AUSNSW	Kocsciousko NP	1
*R. suturalis*	Australia	New South Wales	AUSNSW	Nowra	3
*R. suturalis*	Australia	New South Wales	AUSNSW	Taraga	1
*R. suturalis*	Australia	New South Wales	AUSNSW	Wollongong	2
*R. suturalis*	Australia	Queensland	AUSQLD	Agnes	2
*R. suturalis*	Australia	Queensland	AUSQLD	Bundaberg	1
*R. suturalis*	Australia	Queensland	AUSQLD	Gladstone	3
*R. suturalis*	Australia	Southern Australia	AUSSA	Adelaide	1
*R. suturalis*	Australia	Southern Australia	AUSSA	Mt. Gambier	3
*R. suturalis*	Australia	Southern Australia	AUSSA	Meadows Creek	2
*R. suturalis*	Australia	Southern Australia	AUSSA	Penola	6
*R. suturalis*	Australia	Southern Australia	AUSSA	Robe	1
*R. suturalis*	Australia	Tasmania	AUSTAS	Geeveston	3
*R. suturalis*	Australia	Tasmania	AUSTAS	Togari	1
*R. suturalis*	Australia	Victoria	AUSVIC	Kyneton	2
*R. suturalis*	Australia	Victoria	AUSVIC	Tooborac	1
*R. suturalis*	Australia	Western Australia	AUSWA	Cataby	1
*R. suturalis*	Australia	Western Australia	AUSWA	Manjimup	1
*R. suturalis*	Australia	Western Australia	AUSWA	Manypeaks	1
*R. suturalis*	Australia	Western Australia	AUSWA	Northcliffe	1
*R. suturalis*	Australia	Western Australia	AUSWA	Pilbara	1
*R. suturalis*	Australia	Western Australia	AUSWA	Yanmah State For.	1
*R. suturalis*	Belarus	Minsk Oblast	BEL	Minsk	1
*R. suturalis*	Czech Republic	Liberec	CZE	Liberec	1
*R. suturalis*	France	New Caledonia	NEWCAL	Mt. Mou	1
*R. suturalis*	France	New Caledonia	NEWCAL	Poindimié	1
*R. suturalis*	France	New Caledonia	NEWCAL	Pouembout	1
*R. suturalis*	Indonesia	Flores	INDFLO	Mt. Ranaka/Ranamese Lake	6
*R. suturalis*	Indonesia	Java	INDJAVA	Dieng Plateau	6
*R. suturalis*	Indonesia	Lombok	INDLOM	Sumbalun Lawang	4
*R. suturalis*	Indonesia	Sulawesi	INDSUL	Malino	4
*R. suturalis*	Indonesia	West Sumatra	INDSUM	Danau di Atas	2
*R. suturalis*	Indonesia	Timor	INDTIM	Mt. Mutis	5
*R. suturalis*	Japan	Hokkaido	JAP	Tomakomai	1
*R. suturalis*	New Zealand	Auckland	NEWZEA	Auckland	1
*R. suturalis*	New Zealand	Nelson	NEWZEA	Canaan	3
*R. suturalis*	New Zealand	Southland	NEWZEA	Key Summit	1
*R. suturalis*	Papua New Guinea	Central	PNGCEN	Myola	2
*R. suturalis*	Papua New Guinea	Eastern Highlands	PNGEHP	Aiyura	3
*R. suturalis*	Papua New Guinea	Enga	PNGENG	Wabag	1
*R. suturalis*	Papua New Guinea	Madang	PNGMAD	Mts. Finisterre	3
*R. suturalis*	Papua New Guinea	Sandaun	PNGSAN	Mianmin	4
*R. suturalis*	Papua New Guinea	Sandaun	PNGSAN	Telefomin	1
*R. suturalis*	Papua New Guinea	Southern Highlands	PNGSHP	Mt. Ambua	4
*R. suturalis*	Papua New Guinea	Western Highlands	PNGWHP	Giluwe	2
*R. suturalis*	Papua New Guinea	Western Highlands	PNGWHP	Mt. Hagen Town	2
*R. suturalis*	Papua New Guinea	Western Highlands	PNGWHP	Mt. Hagen Kumul Lodge	2

Specimens of closely related *Rhantus* species, that is, *R. bacchusi*, *R. elisabethae*, *Rhantus* new species 1 and 2, as well as *R. suturalis* from the northern clade were included as outgroups (Balke 2001; Balke et al. 2007, 2009).

Genomic DNA was extracted from legs or thoracic tissues using the DNeasy kit (Qiagen, Hilden, Germany). We sequenced 1095 bp from the mitochondrial cytochrome *c* oxidase subunit 1 (702 bp) and cytochrome b (393 bp) using the primers listed in Table 2 to conduct PCR reactions with standard protocols (http://zsm-entomology.de/wiki/The_Beetle_D_N_A_Lab). Both strands of the PCR products were then sequenced and sequences corrected and aligned using Geneious 5.6.5 (available from http://www.geneious.com) before being exported under Mesquite 2.75 (available from http://www.mesquiteproject.org) to check the reading frame and create three different datasets (CO1, CytB, and *Combined*). We sequenced fragments of the nuclear genes 18S and arginine kinase for several specimens of the southern clade, but no molecular variation in the alignment was identified (data not shown). This is in line with Balke et al. (2009) who used 18S rRNA, wingless, elongation factor 1 alpha (2 exons and 1 intron), and histone 3 and found little or no informative signal within the southern clade. Because of this lack of informative sites, we use fast evolving mitochondrial markers here. All the sequences used in this study are deposited in Genbank under the accession numbers KC604111 - KC604412.

**Table 2:** Primers used to amplify regions of the cytochrome oxidase subunit 1 (CO1) and cytochrome B (CytB)

Locus	Primer	Primer sequence	Reference
CytB	CB3	GAG GAG CAA CTG TAA TTA CTA A	Barraclough et al. 1999
	CB4	AAA AGA AA(AG) TAT CAT TCA GGT TGA AT	Barraclough et al. 1999
Cox1	Pat	TCC AAT GCA CTA ATC TGC CAT ATT A	Simon et al. 1994
	Jerry	CAA CAT TTA TTT TGA TTT TTT GG	Simon et al. 1994

### Phylogeny

Different methods of phylogenetic inference were used for the *Combined* dataset to infer relationships of individuals within *R. suturalis*: (1) distance analyses using the Neighbor-Joining method implemented in Geneious 5.6.5 (Drummond et al. 2012) with 10000 bootstrap replicates and a HKY model of evolution (see below for a rationale on this setting); (2) Maximum Parsimony (MP) analyses using TNT 1.1 (Goloboff et al. 2008) with the Sectorial Searches, *Tree Ratchet, Tree Fusing* and *Tree Drifting* algorithms (Goloboff 1999), and 100 random additional sequences. A Symmetric Resampling with a probability fixed to 10 and 1000 replicates was performed as it allows avoiding uninformative characters, character weight, and transformation costs to affect the resampling unlike classic *Bootstrapping* and *Jacknifing*; (3) Maximum Likelihood (ML) analyses were performed with 1000 bootstrap replicates under RAxML (Stamatakis 2006) with different partitioning strategies: *NoPart* (no partitioning), *ByGene* (one partition for each gene), *ByCodon* (one partition for each codon position), and BySix (one partition for each codon position of each gene); and finally, (4) Bayesian Inference (BI) analyses were performed using the same strategies of partitioning, under MrBayes 3.1.2 (Ronquist and Huelsenbeck 2003). The datasets were analyzed with two independent runs consisting of eight Markov Chains Monte Carlo (MCMC, one cold and seven incrementally heated) sampling for 30 million generations. In order to compute support information, the trees were sampled every 1000 generations and each MCMC started from a random topology. The split-frequencies as long as the log-likelihood curves were investigated to provide a good estimate of the burn-in fraction. Once these samples were discarded, the remaining topologies were used to yield a 50% majority rule consensus tree. Best-fitting partitioning strategies for the ML and BI analyses were selected using Bayes Factors (BF; Kass and Raftery 1995) approximated under Tracer 1.5 (Rambaut and Drummond 2007). The substitution models of evolution for each partition used in ML and BI analyses were selected under jModelTest 0.1.1 (Posada 2008), using the Bayesian information criterion (BIC) rather than the corrected Akaike information criterion (AICc) as advocated by Brown and Lemmon (2007). The HKY model was set for the NJ analysis as it is the closest one to the model selected for the *Combined* dataset implemented in Geneious 5.6.5.

### Phylogeography and historical demography

The phylogeographic pattern within the southern clade was analyzed for all the *Combined* dataset through haplotype network inferred from the 133 specimens of the southern clade in addition to 3 specimens of the northern clade included as outgroups. The sequences were collapsed into haplotypes under DnaSP 5.10 (Librado and Rozas 2009) and a network was inferred using Hapstar 0.7 (Teacher and Griffiths 2011) based on the connection lengths obtained in Arlequin 3.11 (Excoffier et al. 2005).

Historical demography was investigated with the southern clade specimens only using Tajima's *D*, Fu's *F*s*,* and Harpending's raggedness index (Hri). Tajima's *D* (Tajima 1989) and Fu's *F*s (Fu 1997) statistics were calculated using Arlequin 3.11 (Excoffier et al. 2005) with 10,000 permutations to assess whether the mitochondrial data shows evidence of deviation from the neutral theory of molecular evolution holding that stochastic processes such as molecular drift and mutation explain most of the genetic variation found in living organisms. In addition, these statistics can unveil demographic events such as population expansion (significant negative values) or contraction (significant positive values) (Tajima 1989; Fu 1997). The Harpending's raggedness index (Hri, Harpending 1994) based on mismatch distributions, was calculated using 1000 bootstrap replicates to investigate whether the population deviates from a sudden expansion model (Schneider and Excoffier 1999). A significant Hri (*P* < 0.05) indicates a poor fit to the model and therefore does not support a sudden demographic expansion (Harpending 1994).

The magnitude of historical demographic events was investigated using Bayesian Skyline Plots (BSP, Drummond et al. 2005) as well as Extended Bayesian Skyline Plots (EBSP, Heled and Drummond 2008) under BEAST 1.7.4 (Drummond et al. 2012). BSPs allow the inference of population historical demography in a Bayesian framework based on a coalescent model of evolution. The EBSPs are a slightly different method based on BSPs permitting the analyses of multiple loci separately (see Ho and Shapiro 2011 for a review). The.xml files were created with a partition for each gene and the respective models of evolution set according to the results obtained in jModelTest (Posada 2008). The applicability of a molecular clock was tested using PAUP* (Swofford 2003), and as the molecular clock hypothesis was not statistically supported (*P* < 0.05), we used a relaxed clock that allows rate variation among lineages. Therefore, *The Coalescent: Bayesian Skyline* and *Extended Bayesian Skyline* models were implemented with an estimated relaxed clock (uncorrelated lognormal) based on the rate of evolution calculated by Balke et al. (2009) regarding the evolution of the *R. suturalis* complex, including the 95% confidence interval (*r* = 0.019, 95% interval *I* = 0.011–0.028). The rate was set under a normal distribution with the following parameters: initial value = 0.0195, mean = 0.0195, and SD = 0.00435. Two distinct runs of 50 million generations sampled every 1000 generations were performed for each model (BSP or EBSP). After discarding 10% of the samples as burn-in, the convergence of runs was assessed according to the ESS (Effective Sample Size) criterion and the plots were inferred under Tracer 1.5 (Rambaut and Drummond 2007) for the BSP and a graphic program for the EBSP.

### Estimation of divergence times

As the fossil record is scarce for water beetles, and this study focuses on inter- as well as intra-specific levels, we chose to use the previously introduced evolutionary rate (Balke et al. 2009) with different models and parameters as advocated by previous studies (e.g., Ho and Phillips 2009) to infer diversification ages. A.xml file based on the *Combined* dataset was created with the following non-default settings and priors: the *Site Model* was chosen according to the models of evolution used in the phylogenetic analyses and the MCMC parameters were fixed to 30 million generations with sampling every 1000 generations and the first 25% discarded as burn-in. Divergence time analyses were carried out using BEAST 1.7.4 (Drummond et al. 2012) and were performed under both the *Coalescent: Constant Size* and *Speciation: Birth-Death models*. We used estimated relaxed clock rate (uncorrelated lognormal) with a normal distribution (initial value = 0.0195, mean = 0.0195, standard dev = 0.00435), and also a uniform distribution (initial value = 0.0195, upper = 0.028, lower = 0.011). The best topology obtained in BI for the *Combined* dataset was fixed as the reference topology for divergence time estimates by editing the.xml file manually. At the end of each analysis, a 50% majority rule consensus tree was created under TreeAnnotator 1.7.4. Likelihood scores and Bayes Factors (BF) were then calculated under Tracer 1.5 (Rambaut and Drummond 2007) to select the best analysis.

## Results

### Phylogenetic relationships

We obtained fragments of 524–702 bps length for CO1 and 281–393 bps for CytB for 133 specimens to produce an alignment of 1905 bps without stop codons or frame-shift mutations. The best-fitting evolutionary models for each partition are given in Table 3, and a phylogenetic hypothesis is shown in Figure 4, based on the best BI topology for the *Combined* dataset after selection under the BF criterion (Table 4) (for the best topologies recovered in NJ, MP, and ML, see Figs S1, S2, and S3 of the electronic supplementary materials, respectively).

**Table 3:** Selection of the best-fitting models of sequence evolution under the corrected Akaike (AICc) and Bayesian (BIC) information criterions

Dataset	AICc	BIC
*Combined*	TrN + I + G	TrN + I + G
*Combined Position 1*	K80 + I+G	TrN + I + G
*Combined Position 2*	JC	F81
*Combined Position 3*	HKY + G	TrN + G
*Cytochrome oxidase 1*	TrN + I + G	TrN + I + G
*Cytochrome oxidase 1 Position 1*	K80 + I+G	TrN + I + G
*Cytochrome oxidase 1 Position 2*	JC	F81
*Cytochrome oxidase 1 Position 3*	TrN + G	TrN + I + G
*Cytochrome oxidase B*	HKY + I + G	HKY + I + G
*Cytochrome oxidase B Position 1*	TrN + G	TrN + G
*Cytochrome oxidase B Position 2*	F81	F81
*Cytochrome oxidase B Position 3*	HKY + I + G	HKY + I + G

**Table 4:** Best-fitting strategies of partitioning for the BI and ML phylogenetic inferences with Bayes Factors (*B*_F_) estimates, BI harmonic means, and ML optimization likelihoods

			Bayes factors (*B*_F_)			
			
Partitioning scheme	MrBayes harmonic mean	RAxML likelihood	NoPart	ByGene	ByCodon	BySix
Combined NoPart	−6317.20	−5875.28	–	0	0	0
Combined ByGene	−6390.39	−5862.60	>10	–	0	0
Combined ByCodon	−6197.00	−5526.02	>10	>10	–	0
Combined BySix	−6184.51	−5471.57	>10	>10	>10	–

Within the *R. suturalis* species complex, most of the internal nodes were well to strongly supported by bootstrap (BS ≥ 80) or posterior probability (PP ≥ 0.90) values. The monophyly of *R. suturalis* species complex labeled “C1” in Figure 4 was always retrieved (BS = 100/PP = 1.0). The next clade (C2) contains the southern clade and specimens from Sumatra, which are recovered as sister group of all remaining specimens. This clade was retrieved in all methods of inference except in the ML analysis in which they were the first branch of C1.

The next clade (C3) is the southern clade of *R. suturalis* of Balke et al. (2009), here always recovered with strong support (BS ≥ 98/PP = 1.0). *Rhantus ekari* and *R. dani* form clade C4 (BS ≥ 89/PP = 0.99) as the sister group of all the remaining specimens from the southern *R. suturalis* clade (C5). The next clade, C5, recovered in all analyses (BS ≥ 79/PP = 1.0) but in NJ, comprises the clades C6 (*R. riedeli*, *R. supranubicus,* and several specimens of *R. suturalis* from Papua New Guinea and New Zealand), and C7 (with all remaining specimens of *R. suturalis*, mainly from across Australia).

Strikingly, the Papua New Guinean specimens in C6 (all from Mt. Hagen-Ambua highlands region) were recovered in a well-supported, monophyletic, and genetically well-separated clade, the same is true for the New Zealand specimens recovered as the sister taxa of *R. riedeli* in clade C6. This means that there are two clades with specimens that are morphologically *R. suturalis* that group among narrow endemics, morphologically moderately to strongly divergent from *R. suturalis*, and these two clades are genetically isolated from the main clade of morphological *R. suturalis* specimens (C7).

Overall, the topology discloses a striking, partial lack of geographic structure. Exceptions are *R. ekari*, *R. dani*, *R. riedeli,* and *R. supranubicus* endemic to different West New Guinean highland regions and recovered as strongly supported monophyletic clades (BS ≥ 95/PP = 1.0), as well as the Papua New Guinea and New Zealand specimens in clade C6. Australian specimens are scattered in clade C7 without geographic signal, whereas specimens from Papua New Guinea (PNG) are found in small and scattered internal groups in C7, the ones from Eastern Highlands (Aiyura) and Huon-Papuan Peninsula being monophyletic (Fig. 5). Furthermore, Flores as well as Javanese, Lombok, New Caledonian, New Zealand, Sulawesi, or Timorese individuals were recovered as paraphyletic, or in poorly supported clusters.

### Phylogeography and historical demography

**Figure 6:**
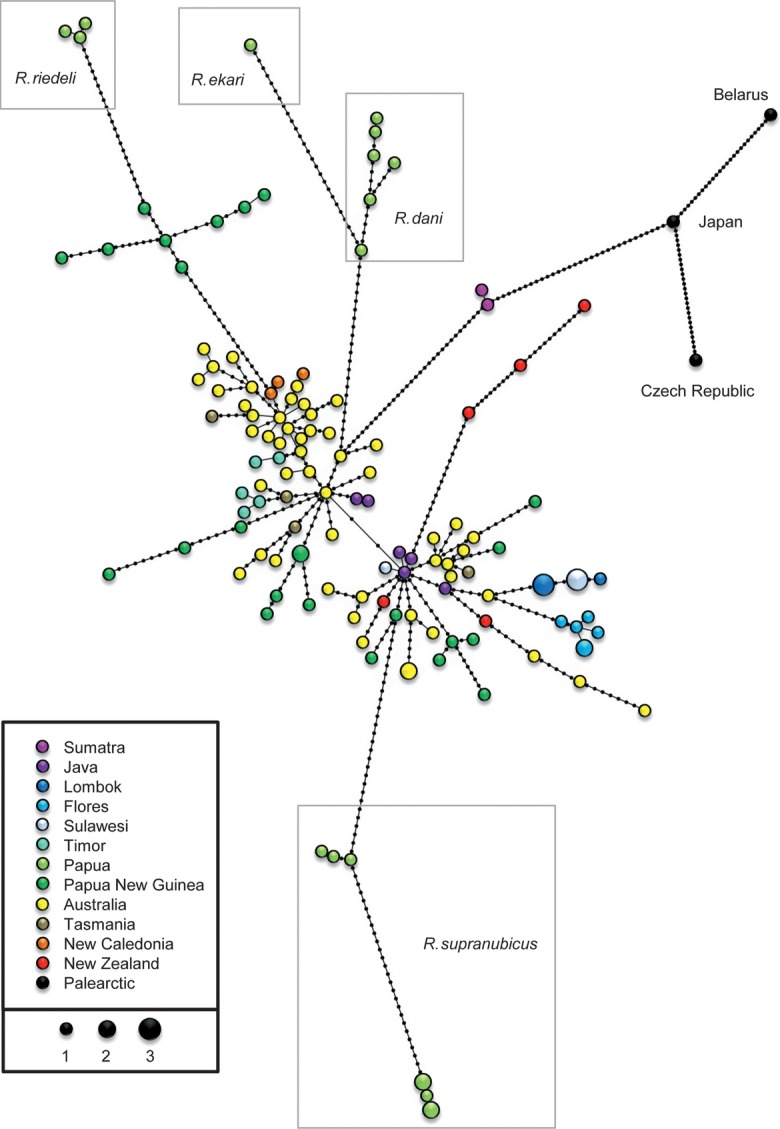
Network based on the *Combined* dataset. The locality and the number of specimen(s) are indicated according to the caption. The black dots indicate missing haplotypes.

All DNA matrices present striking haplotype diversities within the southern clade of *R. suturalis*, from 81% in the CytB to 93% in the *Combined* dataset, these results being supported by high nucleotide diversities (Table 5). Phylogeographic analyses based on the *Combined* dataset yielded a complex network with multiple haplotype series (Fig. 6). The species *R. dani*, *R. ekari*, *R. riedeli,* and *R. supranubicus* from the highlands of Papua New Guinea are well separated from the two central groups of haplotypes formed by numerous specimens of *R. suturalis* mainly from Australia, as well as from Flores, Java, Lombok, New Caledonia, New Zealand, Papua New Guinea, Sulawesi, and Timor. Among these central groups, there is no predominant haplotype and there is a close connection between haplotypes from the entire sampling area; however, no clear geographic structure is recovered, except for the specimens from Flores, which constitute a unique geographic cluster (Fig. 6). This pattern of geographic mixture is less well recovered within highland specimens from Papua New Guinea and specimens from New Zealand, that tend to represent distinct genetic entities similar to the subordinated, formally named Papuan species.

**Table 5:** Genetic structure of each marker and results of demographic index calculations

Dataset	*Cytochrome oxidase 1*	*Cytochrome B*	*Combined*
Length (bp)	702	393	1095
Mean number of pairwise differences	13.25 ± 6.00	6.37 ± 3.04	19.61 ± 8.73
Nucleotide diversity (Pi)	0.0219 ± 0.01	0.0241 ± 0.01	0.0225 ± 0.01
Number of haplotypes	107	106	122
Tajima's *D*	−1.201 NS (0.09)	−0.648 NS (0.30)	−1.068 NS (0.12)
Fu's *F*s	−24.102 *** (0.0008)	−24.987 *** (0.0000)	−23.883 *** (0.0001)
Harpending's raggedness index	0.00320 NS (0.63)	0.00654 NS (0.96)	0.00174 NS (1.00)

NS indicates not significant values. *** indicates highly significant values.

**Figure 7:**
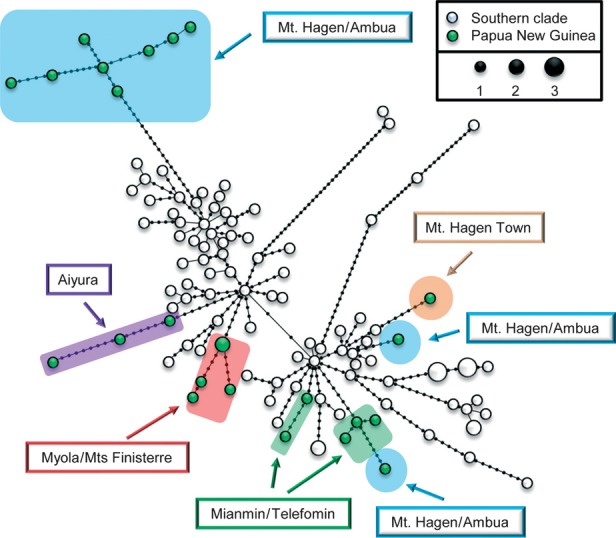
Simplified network based on the *Combined* dataset highlighting the New Guinean specimens. The locality and the number of specimen(s) are indicated according to the caption. The black dots indicate missing haplotypes.

Eight individuals of *R. suturalis* from different high mountains in the Eastern Papua New Guinea highlands (Fig. 7) form a well-delineated clade. Strikingly, one specimen from the valley at the foot of Mt. Hagen, from Mount Hagen town, is in the main haplotype group in one of the star-bursts, and even more strikingly, so are two specimens collected from the high altitude Mt. Ambua locality, taken from the same pool as the specimens in the genetically well-delineated Mt.Hagen-Ambua clade (Fig. 7). These two Ambua specimens cluster with specimens from more western PNG-Papuan highlands localities (Min area in PNG west into Indonesian Papua in the Aipo area) (Fig. 5). Min area specimens are, however, also in a second, geographically proximate clade as well (Fig. 4). PNG specimens from Aiyura, Eastern highlands, form their own clade, as do the Huon Peninsula and Papuan Peninsula specimens. *R. suturalis* specimens from the northern clade are retrieved with a deep genetic divergence highlighted by very long connecting branches, and a geographic continuum, as the Sumatran specimens are more closely related to the southern clades than the Palearctic ones.

The non-significant negative values of Tajima's *D* obtained for all datasets suggest a demographic expansion statistically supported by highly significant negative values of Fu's *F*s. The hypothesis of population expansion is supported by Harpending's raggedness index as well, as the values for all datasets are very low and non-significant (Table 5).

**Figure 8:**
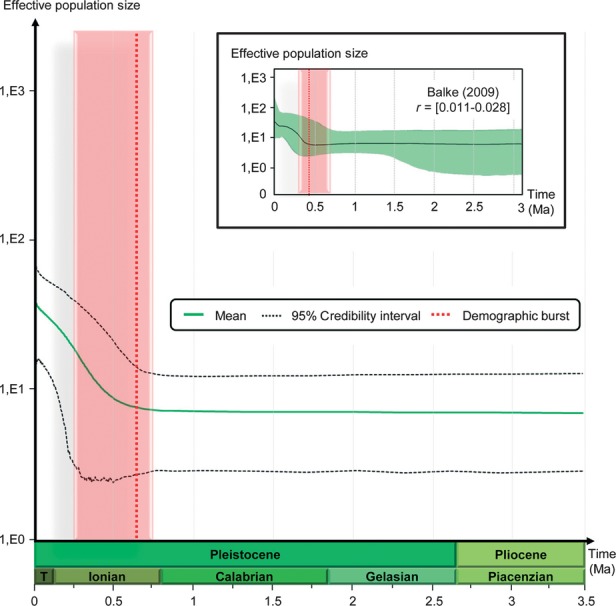
Extended Bayesian Skyline Plot based on the rate calculated by Balke et al. (2009). A 500-kyr timescale is shown at the bottom of the chronogram and spans a period of time from the late Pliocene to the present. Result of the Bayesian Skyline Plot is given in the right part of the figure. Demographic expansion and 95%HPD are shown according to the caption.

All the ESBP and BSP runs converged well according to the log-likelihood curves and ESS checked under Tracer 1.5 (Rambaut and Drummond 2007). The EBSP analysis based on the rate of Balke et al. (2009) highlights a scenario divided into a phase of constant population size, followed by a sudden demographic expansion, likely starting approximately 600,000 years ago during the Late Pleistocene (Fig. 8). The results of the BSP analysis highlight a demographic expansion as well (Fig. 8), with a later approximate age of 450,000 years ago.

### Estimation of divergence times

**Figure 9:**
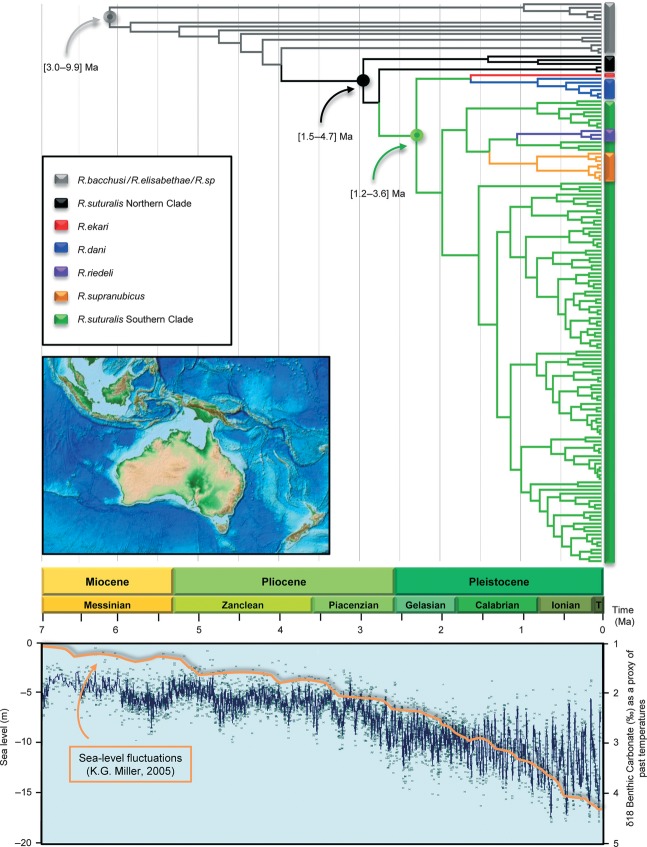
Maximum credibility tree with mean ages (Ma) from the BEAST analysis. A 1-Ma timescale is shown at the bottom of the chronogram and spans a period of time from the late Miocene to the present. The 95%HPD intervals of divergence times are shown between square brackets for the three major nodes of the chronogram. The vertical bands and pastilles at the nodes of different colors referring to the color of the clades highlight groups of interest for which the names are provided in the top left of the figure. A map with bathymetric information (light blue indicates shallow sea/dark blue indicates deep sea) is shown along with a graphic presenting the evolution of sea level and temperature during the last 7 Ma.

The analysis based on the rate of Balke et al. (2009) optimized with a normal distribution and a *Birth-Death model* of speciation was selected under the BF, ESS, and likelihood criterions as the most likely, and the chronogram derived from this analysis is presented in Figure 9. The divergence time estimates obtained for the two best runs (*BDBalkeNorm* and *BDBalkeUni*, see Table 6 for abbreviations) were highly similar with a maximum divergence of less than 4% (∼0.2 Ma) for the mean age of the root (Table 6). Our results show that the most recent common ancestor (MRCA) of the *R. suturalis* southern clade originated approximately 2.3 Ma ago (95% credibility interval: 1.2**–**3.6 Ma) during the Pliocene–Pleistocene transition. Interestingly, most of the intra-specific nodes within the southern clade radiation are young, with ages spanning a period of time from the Early Calabrian (∼1.7 Ma) to the Tarantian (∼100 kyr). The node C4 (*R. dani* and *R. ekari)* is dated to the Early Calabrian (∼1.7 Ma), approximately the same age as the clade C6 (*R. riedeli*, *R. supranubicus*, the Papua New Guinean Mt. Hagen-Ambua clade, and one clade including some of the New Zealand specimens).

**Table 6:** Mean ages (in Ma) and 95% credibility intervals for the different analyses

	Root	C1	C2	C3	C4	C5	C6	C7
1.*BDBalkeN*	**6.1 (3.0–9.9)**	**3.0 (1.5–4.7)**	**2.8 (1.4–4.4)**	**2.3 (1.2–3.6)**	**1.6 (0.6–2.8)**	**2.0 (1.0–3.1)**	**1.7 (0.8–2.7)**	**1.5 (0.8–2.4)**
2.*BDBalkeU*	5.9 (3.0**–**10.1)	2.9 (1.5**–**4.8)	2.7 (1.3**–**4.5)	2.2 (1.1**–**3.7)	1.6 (0.6**–**2.8)	1.9 (1.0**–**3.2)	1.6 (0.8**–**2.8)	1.5 (0.7**–**2.5)
5.*COALBalkeN*	8.8 (4.0**–**14.9)	3.8 (1.8**–**6.2)	3.5 (1.7**–**5.7)	2.8 (1.3**–**4.6)	1.9 (0.6**–**3.5)	2.4 (1.1**–**3.9)	2.0 (1.0**–**3.3)	1.7 (0.8**–**2.9)
6*.COALBalkeU*	9.9 (4.4**–**17.1)	4.1 (2.0**–**6.8)	3.7 (1.8**–**6.3)	3.0 (1.4**–**5.0)	2.1 (0.7**–**3.7)	2.5 (1.2**–**4.2)	2.1 (1.0**–**3.6)	1.8 (0.8**–**3.1)

BD, *Birth-Death* model; COAL, *Coalescent* model; N, Normal law of distribution with uncorrelated lognormal clock model; U, Uniform law of distribution with uncorrelated lognormal clock model.

The text of the best run based on BF, ESS, and likelihood criterions is bold.

## Discussion

### Phylogenetic relationships

We retrieve relationships congruent with the multi-gene mtDNA and nDNA study of Balke et al. (2009), with a robustly supported paraphyly of *R. suturalis*. The split between the northern and southern clades is also recovered here even though our placement of specimens from Sumatra is ambiguous, probably due to the scarce sampling for the northern clade. We find a lack of resolution for most of the *R. suturalis* radiation in the clade C5, except for *R. riedeli*, *R. supranubicus,* and for several individuals from New Guinea and New Zealand, which are grouped in C6 as the sister group of all remaining *R. suturalis* specimens from the entire archipelago. *Rhantus ekari* and *Rhantus dani* are recovered sister species, and they are indeed morphologically similar to each other as well as to *R. suturalis,* whereas the other New Guinea endemics *R. supranubicus* and *R. riedeli* are morphologically (male genital, color, for claws) more deviating. Even though Balke et al. (2009) proposed the inclusion of New Guinean highland specimens of *R. suturalis* among a clade comprising *R. supranubicus* and *R. riedeli*, the placement of specimens from southern New Zealand in this New Guinean clade was highly unexpected. Concerning the New Guinean specimens, the collection localities (remote alpine habitats) seem to indicate that these beetles belong to a well-differentiated population that may represent at least one new putative species. The New Zealand specimens, on the other hand, are recovered in a basal clade that is thought to be the ancestral clade of the *R. suturalis* as recovered by Balke et al. (2009). We suggest that isolated specimens from southern New Zealand are likely a relict population from a first colonization wave through the archipelago, and have most likely been evolving independently from the rest of the radiation for a long period of time. This population from mid-altitude lakes, well-separated from other New Zealand populations, might represent a new species, similar to the New Guinean specimens of the clade C6. More generally, the lack of resolution in C7 and the short branches within the different clades of the topology support a very recent radiation of the southern clade.

### Phylogeographic network

The partial lack of geographic structure seen in the phylogenetic inference is recovered in the haplotype network (Fig. 6). The Papuan species, the Mt. Hagen-Ambua highland specimens from Papua New Guinea and some New Zealand specimens are separated from the core of other haplotypes by a large number of mutational steps. The numerous connections seem to indicate a star-like architecture even though there are some deviations. A star-like network suggests range expansion leading to the evolution of numerous closely related genotypes derived from a wide, central, and often ancestral haplotype (Avise 2000). Here, the lack of one central and widespread haplotype along with the general absence of geographic structuring in the main clades seem to suggest either (1) ongoing but moderate gene flow driven by dispersion within the wider area of distribution, therefore allowing the mixture of genotypes from different localities while avoiding complete homogenization, or (2) the recent cessation of gene flow across the Archipelago, thus leading to the isolation of populations that start to diverge genetically (e.g., Ribera et al. 2011). In the latter case, we suggest that the populations are not clustered in well-distinguished geographic groups because of the recency of the event. Even though there is a lack of clear geographic correlation, all specimens of some localities are closely related (e.g., Flores, New Caledonia, Timor, PNG Telefomin-Min area, PNG Aiyura area etc., Figs 7), indicating the initiation of geographic structuring or colonization from the same or related sources. Interestingly, New Zealand specimens in a distinct clade in the phylogeny are not recovered close to New Guinean specimens, but are connected to a central and unique Javanese haplotype, most likely the result of incomplete lineage sorting. The deep divergence between these specimens and the central haplotype group, including the rest of the specimens from New Zealand, supports our hypothesis of an older isolation and restricted gene flow hinting toward ongoing speciation, a similar pattern as the one observed for the Papuan highland species. In agreement with the phylogenetic inference, the group of specimens from Papua recovered in C6 is separated from the central haplotype group and clusters with *R. riedeli*, while still exhibiting a deep genetic divergence between the two. In addition, most of the specimens from Papua New Guinea are restricted to small groups in the periphery of the phylogeographic networks, suggesting colonization or ongoing geographic isolation from the rest of the populations.

More importantly, it was astonishing to find syntopically occurring Mt. Ambua (Papua New Guinea) specimens in isolated clades C6 and C7 (Figs 7). Specimens in C6 form a well-delineated clade in the network as well as in the phylogenetic trees. The Ambua specimens from the main clade C7 group with individuals from the mountain chain west of the Mt. Hagen-Ambua area, that is, the Telefomin-Mianmin-Aipo area (the wider Star Mountains, Fig. 7 map). The presence of two genetically very distinct populations of *R. suturalis* in the same locality suggests longer isolation of a population in the Mt. Hagen-Ambua area (Figs 7), and recent secondary contact with dispersers out of the widespread clade C7. It is striking to note that the single specimen we obtained from the foot of Mountain Hagen, from the valley close to Mt. Hagen Town, also belongs to the large clade C7 and has no closer relatives, supported in both tree and network inference.

### Pleistocene evolution in the *R. suturalis* complex

The divergence time estimates support an Early Pleistocene origin of the *R. suturalis* southern clade, while the branching pattern indicates that the radiation of the group started more recently, most likely in the Middle Pleistocene (∼1 Ma). Furthermore, the sudden demographic expansion during the late Pleistocene, *c*. 600 kyr ago, corroborates this scenario of recent radiation during the Quaternary ice ages (2.4 Ma until present) (Hewitt 2000). By then, the high mountains of New Guinea and their (peat) swamps existed already, meaning that vast, highly structured highlands and associated habitats were available.

During the last decades, the impact of Pleistocene glaciations on tropical regions has been widely acknowledged, including global cooling, rising aridity, rainforest depletion, and ecosystem fragmentation associated with refugial budding especially in highlands (Hewitt 2000; Hope et al. 2004; Rull 2011). On the other hand, the dispersal or adaptation of taxa driven by habitat loss or alteration has been increasingly highlighted recently for several insect groups, assuming that the type of response to climate shifts lies on the timescale considered (e.g., Smith and Farrrell 2005; Winkler et al. 2009; Hawlitschek et al. 2012; Toussaint et al. 2012). Interestingly, our findings support the hypothesis that diversification and dispersal of *R.suturalis* started during the Plio-Pleistocene transition (∼2.5 Ma), as advocated by Balke et al. (2009). Moreover, the placement of New Guinean species suggests a first transgression of Wallace's line during the early Pleistocene (∼2.4 Ma) followed by a settlement in New Guinean highlands. Dispersal across the Australian region possibly out of New Guinea, toward New Zealand, New Caledonia, Sahul, and Wallacea, with secondary transgression of Wallace's line into Java occurred at the same time as the ongoing cooling of the region in the Pliocene (Figs 9).

During this period, the sharp reduction in temperatures in New Guinean highlands may have led to altitudinal migration implying downward dispersion of most of the species, but also the adaptation of some populations to cooler climate, therefore promoting isolation by vicariance (Rull 2011). At this time, New Guinea had a similar relief to today, with extremely rugged highlands surrounded by lowland tropical rainforests on either side, and often interspersed with chains of lowland forests in-between. Therefore, and even though forest expanse was declining, different geographic localities would have had significantly different microclimates during glacial maxima (Hewitt 2000; Hope et al. 2004), with the likely presence of multiple suitable refugia. Populations trapped in these sky islands then likely evolved in a similar way to the classic case of oceanic island isolation (Gillespie and Roderick 2002), while the downward migration of the New Guinean highland biota and its dispersion in Australasia might have promoted speciation in lowlands. As advocated by Verstappen (1997) and Hewitt (2000), the Pleistocene glaciations, even though driving global cooling, nevertheless constituted a succession of warmer and cooler periods known as the Milankovitch cycles. Therefore, highland populations might have been separated in times of cooling, promoting genetic isolation in sky islands, before being reconnected to other populations during warmer climatic phases.

Interestingly, our results underpin the different expected prospective stages of an early lineage diversification: (1) within the widespread morphologically delineated *R. suturalis*, there are different narrow-endemic species, which are morphologically quite distinct from *R. suturalis* (*R. riedeli*, *R. supranubicus*, with differences in male genitalia and male fore claws, the latter also displaying different coloration); (2) there are different narrow-endemic species with differences in male genitalia, but otherwise rather similar to *R. suturalis* (*R. dani*, *R. ekari,* and *R. kakapupu,* the latter not sequenced here); (3) there are genetically isolated groups morphologically, however, assigned to *R. suturalis* (e.g., the Mt. Hagen-Ambua clade, as well as an isolated New Zealand clade); and (4) within *R. supranubicus*, we find deep divergence between specimens from the same puddles, collected over a decade. The latter case, as well as the syntopical presence of the Mt. Hagen-Ambua clade with individuals from a distant clade, strongly support the idea that isolation of populations after dispersal can be comparably long, but secondary contact of populations might occur at any time.

Here, we suggest that this budding speciation might represent a good example of peripatric speciation, as the populations were not only geographically separated but also had to adapt to local ecological conditions in the periphery of the species distributional range. While this ongoing speciation process occurred in highlands, the Australian lowland populations obviously remained connected by strong gene flow as indicated by the low divergence levels of the haplotypes among the southern clades. Populations in Sunda and the Wallacean mountains, with the exception of Flores, were not monophyletic (Fig. 6), indicating incomplete lineage sorting after very recent arrival, or continuous gene flow across tropical lowland and oceanic barriers. The syntopic occurrence of beetles from distinct clades, as illustrated by New Guinea and New Zealand highland communities, might indicate that these parts of the *R. suturalis* species complex are at the end of an isolation stage. Interestingly, the exclusion of Sumatran specimens from the southern clade confirms the hypothesis of Balke et al. (2009), suggesting that the northern and southern clades are now separated by only a few 100 km between Sumatra and Java.

There might be another interpretation of these macroevolutionary patterns. The northern and southern clades of *R. suturalis* could represent two morphologically highly similar yet different species. We did not consider assigning two species names because we lack samples from southern Sumatra and mainland Southeast Asia and the Philippines, which we suggest might help to better understand species limits in this complex. If there were indeed two distinct species, and depending on the topology of the undersampled northern group, the supertramp trait might either still be ancestral in the *R. suturalis* complex, or has originated twice. In the southern group, this would be the origin of a supertramp (*R. suturalis*, which has its type locality in Java) out of the New Guinea clade of narrow-endemic species. Colonization of the Australasian region would have led to the formation of a paraphyletic series of narrow-endemic species, and then origin of the widespread New Guinea-Australian region-Wallacean supertramp. Peripheral speciation would, in that case, be ongoing in the rather deviating New Guinea and possibly Flores clades within *R. suturalis*.

## Conclusion

*R. suturalis* is a morphologically rather uniform, very widespread, dispersive species and ecological generalist, occurring from saline ponds in oasis up to high altitude peat swamps, although being absent from tropical lowlands. Populations of *R. suturalis* across the Australasian-Indomalayan region are well connected by ongoing dispersal while peripheral speciation processes occur in highland ecosystems. Mountains of New Zealand and much more, so in the extremely rugged vast highlands of New Guinea, apparently harbor different stages of speciation. There are four narrow endemics, morphologically and genetically distinct species emerging from within the widespread genealogy. Furthermore, there are several genetically more or less divergent, isolated clades that agree morphologically, however, with the widespread form. We suggest that reproductive isolation has been shaped by Quaternary glaciations that promoted peripheral budding especially in New Guinea sky island ecosystems. The general cooling since the Pliocene might have promoted the demographic expansion and wide dispersion observed in *R. suturalis*, out of a clade of narrow endemics. *Rhantus suturalis* illustrates the reversal or switch of endemic species to widespread generalists, and then toward narrow-endemism (presumably with loss of physiological tolerance) again. This is an element of the taxon cycle (Wilson 1959, 1961), which predicts that higher physiological tolerance might occur at some stage in lineage evolution and promote colonization of new areas, and ultimately diversify into more specialized habitats. The high mountains of New Guinea act as a diversity pump for the region and therefore represent an evolutionary cradle of diversity, certainly deserving further investigation based on more extensive taxon and character sampling.

## Supporting Information

Figure S1. Best neighbour joining topology.

Figure S2. Best maximum parsimony topology.

Figure S3. Best maximum likelihood topology.
