# Supplementary figures and images for: Australasian sky islands act as a diversity pump facilitating peripheral speciation and complex reversal from narrow endemic to widespread ecological supertramp
Source: Ecol Evol. 2013 Mar 7;3(4):1031–49. doi: 10.1002/ece3.517 (PMC3631412; doi:10.1002/ece3.517)

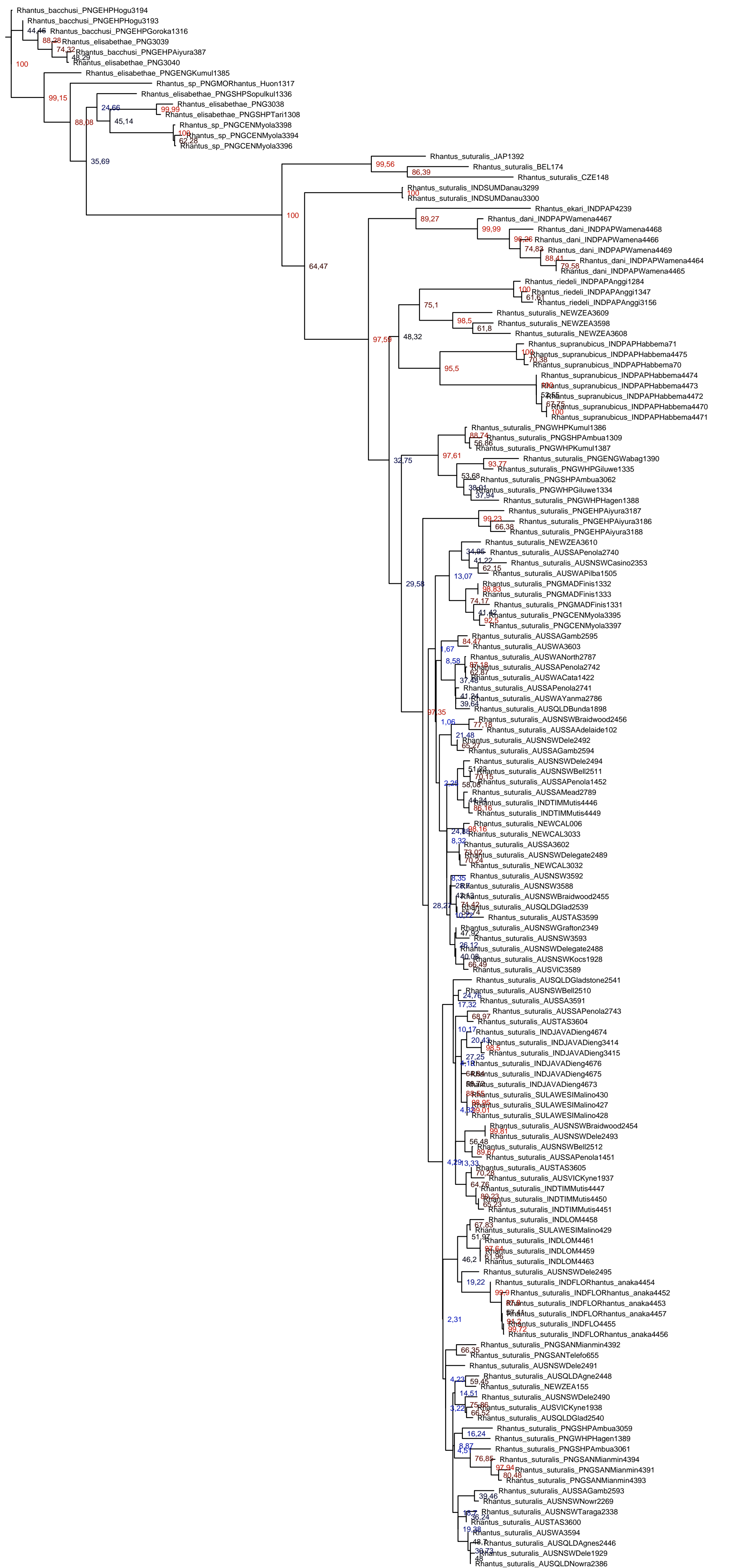

0.01

Supplement: Supplementary file 1 [file ece30003-1031-SD1.pdf]

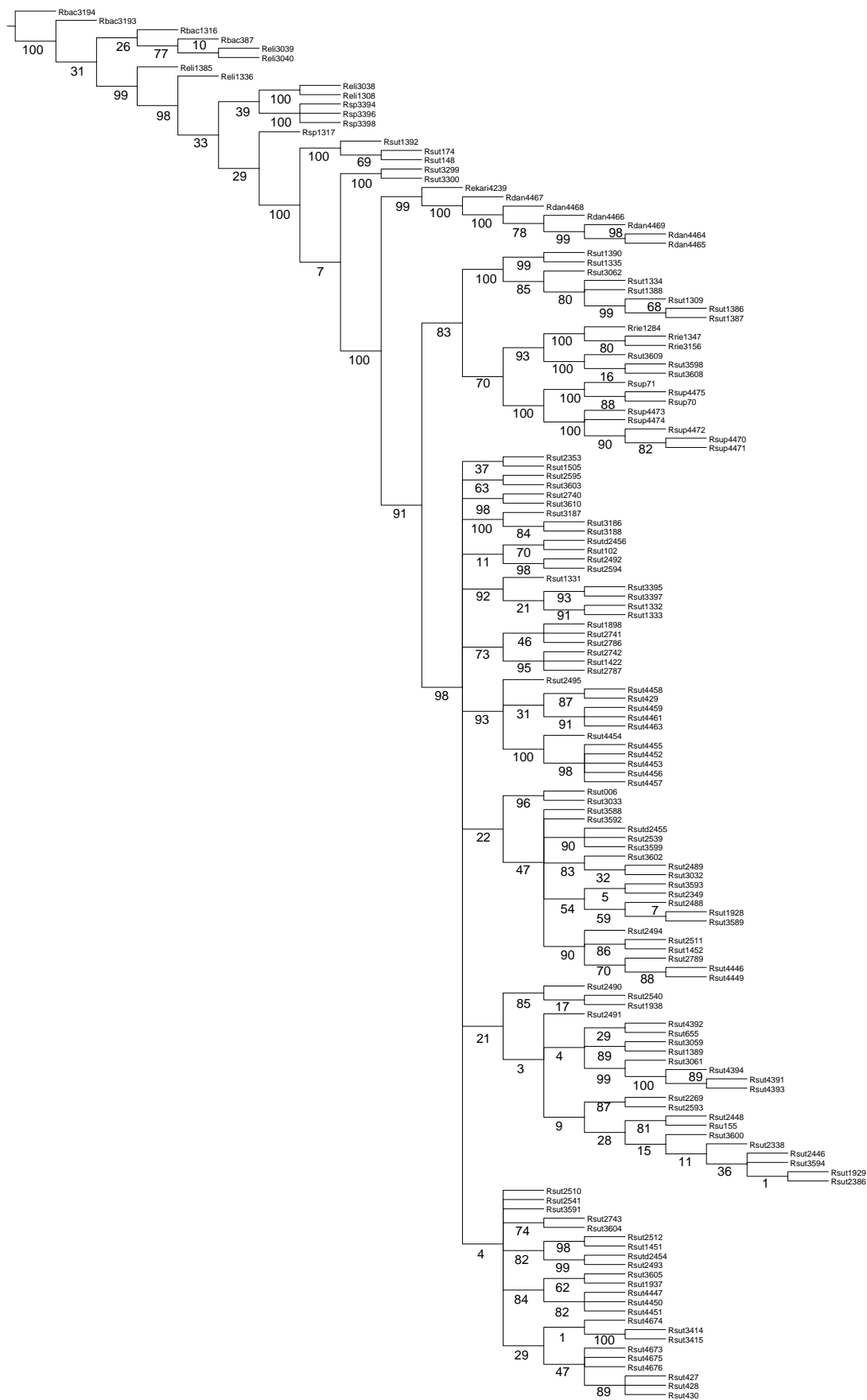

Supplement: Supplementary file 2 [file ece30003-1031-SD2.pdf]

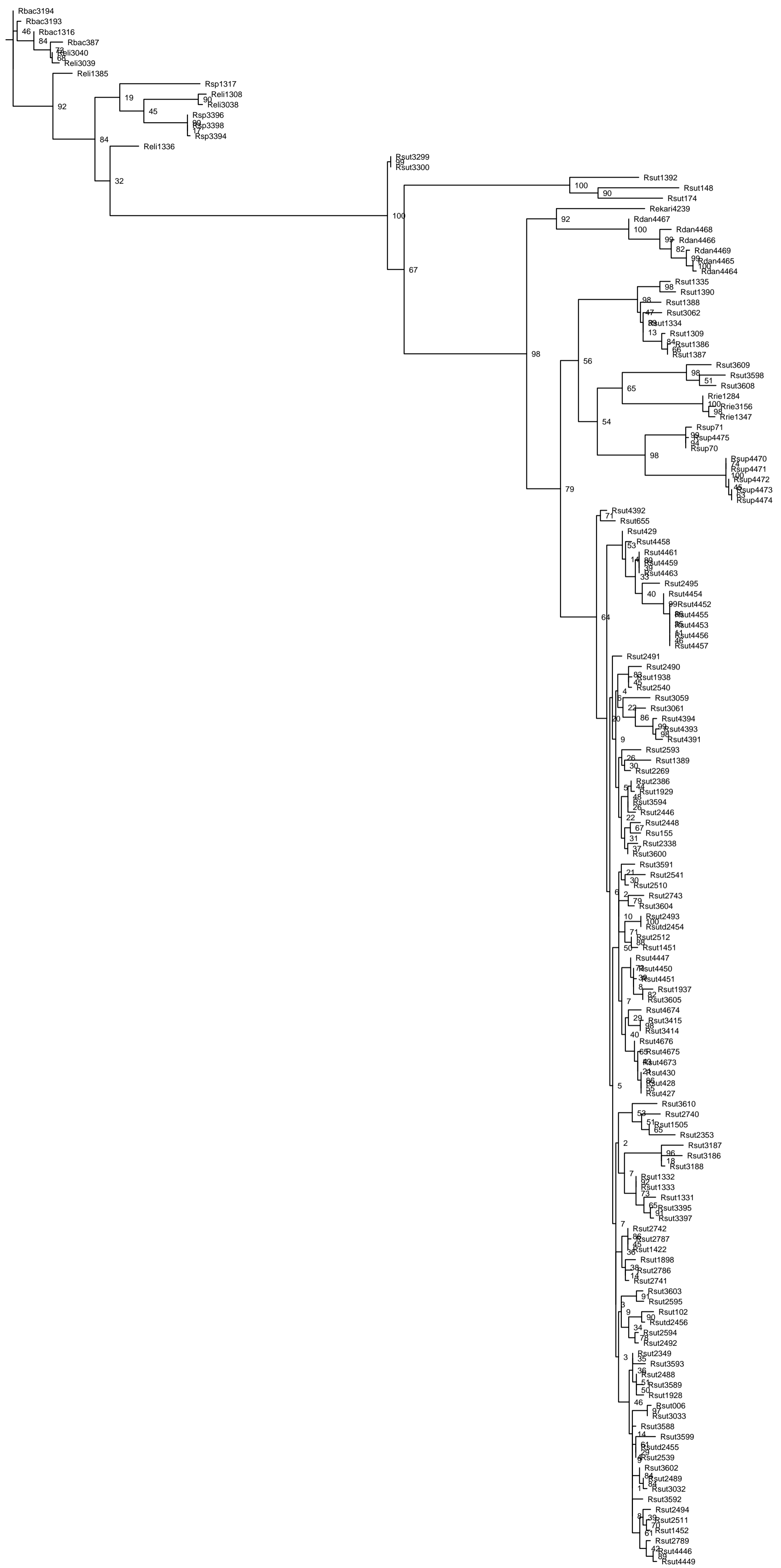

Supplement: Supplementary file 3 [file ece30003-1031-SD3.pdf]
